# Tissue-type Differences in Focused Ultrasound and Microbubble-mediated Drug Delivery to the Brain Exist at Vessel Level

**DOI:** 10.7150/thno.117691

**Published:** 2026-01-01

**Authors:** Alessandro De Maio, Fa-Hsuan Lin, Bojana Stefanovic, Meaghan A. O'Reilly

**Affiliations:** 1 Physical Sciences Platform, Sunnybrook Research Institute, 2075 Bayview Avenue, Toronto, ON M4N 3M5, Canada.; 2 Department of Medical Biophysics, Faculty of Medicine, University of Toronto, Toronto, Ontario, Canada.

**Keywords:** Focused ultrasound, drug delivery, blood-brain barrier opening, brain tumor therapy, white matter therapy

## Abstract

**Rationale:** The efficacy of drug delivery to the brain is constrained by the impermeability of the blood-brain barrier (BBB) in healthy tissues and the heterogeneous permeability of the blood-tumor barrier (BTB) in gliomas. Focused ultrasound (FUS) has emerged as a promising technique to transiently modulate vascular permeability, however its effects vary across different brain tissues. This study systematically evaluates the effects of FUS-induced vascular permeability modulation in the gray matter (GM), white matter (WM), and brain tumors, considering their distinct tissue architectures, vascular densities, and permeability profile. Additionally, we compare the delivery of bevacizumab (antiangiogenic monoclonal antibody) and methotrexate (small-molecule chemotherapeutic) to determine how molecular size influences vascular-level permeability and extravasation distances.

**Methods:** A total of n = 48 Fischer-344 rats, including both healthy and tumor-bearing cohorts, underwent magnetic resonance imaging (MRI)-guided FUS using a feedback-controlled algorithm to modulate microbubble pressure based on microbubble emissions. Tumors were either untreated or received a single FUS exposure, while healthy tissues, including GM and WM, were treated with either a single exposure, or a repeated exposure administered 30 minutes after the first one. MR images were used to assess contrast enhancement before and after sonication. Drug deposition was quantified via fluorescence microscopy in terms of local signal intensities and distances of extravasation. Tissue-specific vascular characteristics, including vessel diameters, densities, and inter-vessel distances, were also analysed.

**Results:** The lack of MRI contrast enhancement in untreated tissues suggested a healthy permeability status of the BBB in GM and WM, while a compromised BTB was observed in tumors. Following FUS treatments, contrast enhancement significantly increased in all tissues, with tumors exhibiting the most pronounced effects. Repeated FUS further enhanced permeability in GM and WM, achieving drug deposition levels comparable to those observed in tumors after a single treatment. At the vascular level, FUS exposure led to significant increases in drug extravasation distances, particularly in tumors. Vascular densities were approximately threefold higher in GM, compared to WM and tumors (GM:WM:Tumor 3.2:1:1), yet both drug signal intensities and extravasation distance correlated more strongly with the number of treatments than with baseline vascularity. Fluorescence microscopy revealed that bevacizumab extravasation was primarily localized near vessel lumens, whereas methotrexate exhibited significantly greater extravascular diffusion, reaching distances spanning entire inter-vessel spaces, consistent with its lower molecular weight. At the individual vessel level, white matter showed significantly lower drug signal intensity than gray matter following a single treatment.

**Conclusion:** This study provides vascular-level insights into how FUS-mediated drug delivery is influenced by tissue architecture, vascular properties, treatment regimen, and drug molecular weight. Notably, at the individual vessel level, drug extravasation varies between the different tissue types, and thus vascular density is not the sole driver of differences in drug deposition in these tissues. The study findings highlight the potential of repeated FUS exposures for enhancing the deposition of therapeutics across the physiologically intact BBB of both the gray and white matter, reaching levels comparable to those observed in the pathologically compromised BTB of gliomas. Thus, sonications prescribed over previously permeabilized tissues facilitate deeper drug penetration into interstitial compartments, allowing therapeutics to reach cells further from vessel lumens despite inherent tissue-specific differences.

## Introduction

The brain is a highly protected organ, relying on a specialized vascular network to regulate nutrient and oxygen supply. Its vasculature originates from major peripheral vessels, which progressively decrease in size and complexity, branching to ensure a homogeneous blood distribution based on metabolic demands. Larger vessels with multilayered walls transition into arterioles and venules, where endothelial cells are accompanied by sparse smooth muscle cells and pericytes, ultimately forming capillaries composed of a single endothelial layer. In physiological conditions, molecular exchange between circulating blood and brain parenchyma occurs exclusively at the capillary level, where the blood-brain barrier (BBB) tightly regulates permeability by modulating vascular tone, interstitial fluid pressure, and cellular transport mechanisms 1.

The endothelial cells serve as a physical barrier separating the vascular compartment from the surrounding interstitial spaces where astrocytes, pericytes, microglia and vascular interneurons coordinate responses to insults and neuroinflammation while providing metabolites to support neuronal function 1.

The BBB presents a major challenge for drug delivery, as it prevents the passage of up to 98% of currently available small-molecule drugs, and virtually all macromolecules 2,3. Passive diffusion is thus largely restricted to lipophilic compounds with molecular weights below 400-600 Da. These can cross the BBB either through the endothelial lipid bilayer or the tight junctions 4, specialized endothelial cell contacts that are dynamically regulated to maintain barrier integrity 5. Active efflux transporters further limit drug deposition, expelling unwanted molecules back to the bloodstream thanks to broad substrate recognition 6, significantly reducing the window of therapeutic activity, as observed with small-molecule chemotherapeutics such as doxorubicin 7 and methotrexate 8.

Focused ultrasound (FUS), combined with microbubbles, provides a non-invasive physical method to transiently and reversibly modulate the permeability of the brain vasculature, facilitating the delivery of otherwise impermeable drugs to the brain 9,10. The exact mechanisms underlying such treatments is not entirely understood, however the periodic oscillations in size, or cavitation, of microbubbles exposed to low-intensity ultrasound are believed to disrupt the endothelial tight junctions by stretching blood vessels along their diameter 11-14. Microbubble cavitation also results in locally altered flow of the surrounding medium, with the generation of convection currents, a phenomenon described as acoustic microstreaming 15, while collapsing bubbles can produce fluid jetting 16. These effects physically promote the permeability of the BBB 17,18 while redistributing solutes from the intravascular spaces to the parenchyma 9,19. *In vivo* microscopy studies show that, immediately following ultrasound exposure, the vasculature undergoes brief cycles of vasospasm, then vasoconstriction gradually dominates over vasodilation and is maintained for up to 5-15 min 12,14. Such response mechanisms have been suggested to be protective and ameliorate the FUS-induced increased vessel permeability determining vasogenic edema 20,21.

While FUS shows potential for effective drug delivery to the brain, recent animal studies 22,23 and clinical trials 24,25 suggest the variability in its effectiveness is linked to the underlying tissue architecture. The central nervous system (CNS) is structurally divided into gray matter (GM) and white matter (WM), each characterized by distinct cellular composition, function, and vascular organization. Clusters of neuronal cell bodies at the level of the cortex, basal ganglia and hemispherical nuclei compose the gray matter, site where synaptic activity and signal processing occur, leading to high metabolic demands. Extending from the neuronal cell bodies, their myelinated axons transmit signal between distant GM regions in highly compacted bundles. The energy requirements for the WM are primarily linked to myelination and axonal maintenance, and are 2-4 times lower than for the GM 26,27. These functional differences are reflected in their vascular architecture: more homogeneous in the GM, while sparser, with up to 8 times lower densities in the WM, adapted for the distinct tissue, structural organization and blood flow 28. The blood-brain barrier (BBB) remains tightly regulated across both tissues, however the phenotypic variation in astrocytes present in the WM is associated with higher expressions of tight junction proteins, higher transendothelial electrical resistances, and consequently, lower solute permeability 29,30.

Central nervous system tumors consist of dramatically different tissue architectures, often showing increased cellular density, irregular vascularization, and metabolic niches that vary from necrotic and hypoxic areas with poor perfusion, to hypervascularized regions. The blood-tumor barrier (BTB) differs substantially from the BBB, exhibiting heterogeneous permeability across tumor regions 31, particularly in high-grade gliomas 32. Displaced astrocytes and pericytes, altered tight junction expression and increased transcytosis transport compromise the integrity of the barrier 33. The heightened permeability of the BTB is leveraged in current antineoplastic therapies, enabling even large molecules like monoclonal antibodies to reach cancer cells 34-36. However, their poor efficacy can be explained by the higher interstitial pressures, hindering bulk flow 37, and the often overexpressed efflux pumps 38-40, lowering the effective drug concentrations permeating tumor tissues.

In tumors, FUS-mediated drug delivery has been shown to increase drug deposition, reduce tumor volume, and improve survival in animal models compared to standard drug administration 41-44. Most clinical trials on FUS-mediated modulation of brain vascular permeability are phase I, primarily concentrated on safety and treatment tolerance 43,45. However, FUS has shown clinical promise in temporarily increasing contrast uptake 13,46, fluorescence dye extravasation, and drug deposition 47-49 as evidenced by magnetic resonance imaging (MRI), intraoperative assessment and post-treatment tumor analysis 50.

In healthy tissues, while the permeability of the GM can be reproducibly modulated with FUS, the WM poses unique challenges in terms of tissue organization and vascularization, with inconsistencies observed across both small 51,52 and large animal models 53,54, as well as in humans 24. Repeating ultrasound exposures at short intervals has been proposed as a strategy to further increase the extent of vascular permeability, and therefore increase the levels of drug delivery. As demonstrated in simulation studies 55, and later confirmed in rodents 56,57, such a technique may provide a safer alternative to higher ultrasound energy or bubble concentrations, with reproducible modulation of the BBB also in the WM at a macroscopic level 23.

While neurodegenerative disorders characteristically affect the GM 58,59, retrograde damage manifests in the WM with microstructural alterations and disorganized action potentials 60**.** Psychiatric illnesses 61,62 and demyelinating disorders like multiple sclerosis 63 are predominantly associated with insults to the WM circuits. Brain tumors can emerge from either of these two structures based on their cellular origins, however the WM represents a niche of preferential development and invasion for high-grade gliomas 64,65**,** rapidly dividing and hijacking the surrounding healthy tissue, leading to poor prognosis despite treatment attempts. It is thus essential to ensure optimal drug delivery irrespectively of tissue-type.

Building on this, the present study investigates feedback-controlled FUS-mediated delivery of both small- and large-molecule therapeutic agents across the BBB of physiological tissues (GM and WM) as well as the BTB of gliomas. The aim is to assess drug extravasation across three distinct barrier permeability conditions: impermeable, characteristic of physiological GM and WM; single permeable, observed in healthy tissues following a single FUS treatment and in untreated tumors; and double permeable, found in healthy tissues receiving repeated FUS exposure and in tumors receiving single treatment. In particular, through vessel-level analysis we seek to determine if differences in drug delivery in response to FUS can be primarily explained by differences in vascular density across tissue types.

## Methods

### General animal care

Following the guidelines provided by the Canadian Council on Animal Care, all animal procedures, including brain surgeries and image-guided experiments were approved by the Animal Care Committee at Sunnybrook Research Institute. General animal care included housing on a reverse light cycle at the Sunnybrook Research Institute animal facility (Toronto, ON) with access to food and water *ad libitum*.

### Experimental design

To investigate the effects of FUS-mediated vascular permeability modulation on gray matter, white matter and tumor tissue, a total of 48 Fischer-344 (males and females, 200-250g, 50-70d) rats were equally allocated into eight cohorts based on the pathophysiological brain condition, administered drug and treatment scheme **(Table 1)**.

**Figure 1:**
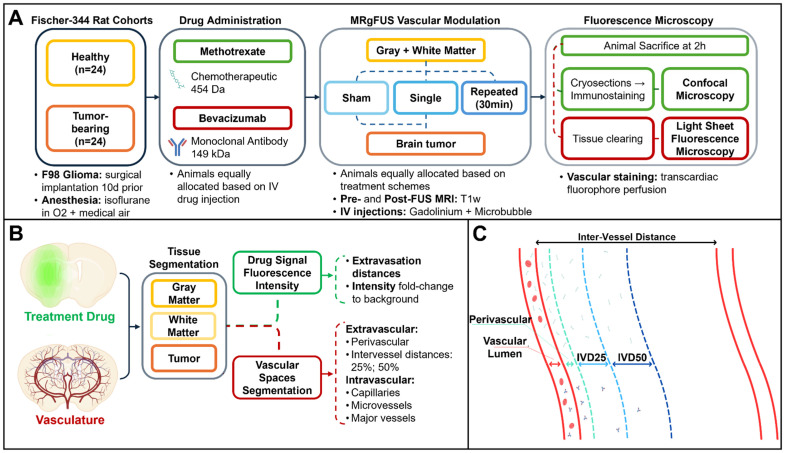
** Overview of methods. (A)** Magnetic resonance-guided focused ultrasound (MRgFUS) timeline. A total of n = 48 Fischer-344 rats were equally allocated according to the underlying tissue architecture (healthy, tumor-bearing), administered antineoplastic (methotrexate, bevacizumab) and FUS treatment (sham, single or repeated exposure). Following MRgFUS, each animal underwent transcardiac perfusion with a dye staining the brain vasculature, then each collected brain was prepared for fluorescence microscopy. **(B)** Following image acquisition, brain tissues were manually segmented based on the tissue autofluorescence, then the treatment drug channel was used to measure intensity values normalized to background, as a way to express drug deposition as an effect of the MRgFUS procedure. On the vasculature channel, vessels were first separated from background, then classified based on their diameter. The ratio of intravascular volumes over the total image sample defined the vascular density of each tissue. Extravascular spaces were instead segmented based on inter-vessel distances **(C)** into perivascular space, 25% of the inter-vessel distance and 50% of the inter-vessel distance in order to measure signal deposition over the entire path of extravasation. Lastly, from the obtained tissue and vessel masks, drug intensities were used to automatically estimate extravasation distances for both antineoplastics.

Animals were distributed into age- and sex-matched healthy (n = 24) and tumor-bearing rats (n = 24) **(Figure 1A)**. The healthy rat cohort (n = 24) was from a previous study 23 and was retrospectively analysed here at higher magnification. The tumor-bearing rat cohort included new animal experiments following similar methodologies, expanding on the aim of the previously presented body of work. The intravenously (IV) injected antineoplastics were: bevacizumab (Avastin, BVZ), an anti-vascular endothelial growth factor (VEGF) monoclonal antibody, and methotrexate (MTX), a folate antimetabolite, prescribed respectively with doses of 50 mg/kg and 30 mg/kg, based on human drug dose equivalents for brain tumor treatments. The fluorescence detection of both drugs was possible by covalently tagging 1mg of bevacizumab with Alexa Fluor™ 647 (Thermo Fischer Scientific, Protein Labeling Kit, Catalog No. A20173) prior to injection *in vivo*, and by immunostaining methotrexate *ex vivo*.

Following previously reported experimental designs 23, healthy rats underwent either single or repeated FUS exposures, targeting the unilateral cortex and putamen for the gray matter, as well as the corpus callosum and internal capsule for the white matter. Tumor-bearing rats, instead, underwent either sham or single FUS sonication over the entire brain tumor volume, as well as adjacent unaffected healthy gray and white matter.

### F98-glioma tumor implantation

F98 glial-like tumor cells were purchased from ATCC (CRL-2397, American Type Culture Collection, USA) at passage number five and cultured following previously described methods 66-68. In brief, cells were cultured in a humidified incubator (5% CO2, 37 °C) in Dulbecco's Modified Eagle Medium (DMEM) supplemented with 10% Fetal Bovine Serum (FBS).

Cell viability was assessed with routine visualization at the microscope and assessed via trypan blue exclusion. Animals scheduled for unilateral tumor implantation were first anesthetized with 2% isoflurane in oxygen, then a C-shaped skin incision was made over the skull exposing the bregma for stereotaxic registration. A 1 mm burr hole was drilled (0.7 mm round Carbide Bur (HM1-007-HP, Meisinger, Germany), in the skull 0.5 mm anterior and 2.8 mm lateral to the bregma (right frontal bone). A 5 μL suspension with a 1:1 ratio of 4 x 10^4^ cells to Matrigel (Corning, Tewksbury, MA, USA) was injected into the caudate putamen (structure of the deep gray matter) 5.0 mm from the dural surface using a 10 μL airtight syringe (Hamilton Company, Reno, USA) attached to a stereotaxic apparatus (David Kopf Instruments, Tujunga, USA). The injection occurred over 1 minute, and the needle was slowly removed after 5 more minutes to allow the matrix to solidify. Lastly, the hole on the skull was covered by bone wax (Ethicon, W31C) and the skin sewn with 3 - 0 braided silk sutures. Animals were then scheduled for MR-guided FUS treatments 10 days after surgery, with sutures removed before FUS.

### Magnetic resonance-guided focused ultrasound treatments

A 7 T magnetic resonance imaging (MRI) scanner (BioSpec 70/30 USR; Bruker, USA) was used for treatment targeting and procedural monitoring by means of serial T1-weighted acquisitions (fast-spin echo: TE = 5.5 ms, TR = 500 ms, 12 averages, matrix: 200 x 200, voxel size: 0.2 x 0.2 x 0.5 mm). A spherically curved lead zirconate titanate (PZT) transducer (75 mm diameter, F#0.8) with 580 kHz fundamental frequency and a co-focused PZT receiver (16 mm diameter, 850 kHz) were mounted on an LP-100 3-axis preclinical FUS system (FUS Instruments, Canada), respectively used for ultrasound delivery and passive acoustic monitoring. The estimated natural focus was 2.7 mm and 22.6 mm respectively for the lateral and axial full widths at half maximum. Exposures, consisting of 10 ms bursts at the fundamental frequency, were administered with a 1 Hz pulse repetition frequency for a total of 2 minutes. Treatment of individual targets were interleaved, with the treatment platform cycling between all targets during each repetition period, such that all targets received 120 bursts within a single 2-minute treatment. Due to mechanical constraints of the motors, if grid size exceeded 3 x 3 then the gid was covered over two treatments, each 2 minutes in duration, allowing 5 minutes between the start of sonications for microbubbles from the previous bolus to clear. Acoustic pressures were actively controlled with a previously described algorithm independently modulating each treatment location 69. In brief, the baseline ultraharmonic signal was recorded following 10 s of 28kPa low pressure pulses, defining a threshold level of 10 times its standard deviation. With the administered microbubbles entering the bloodstream, the algorithm was set to increase the delivered ultrasound pressures by 8 kPa at each pulse repetition (1 s). As the ultraharmonic spectral content reached or surpassed the thresholds determined during baseline recording, the acoustic pressures were reduced by 50% and maintained until either another threshold event occurred (subsequently triggering the pressures to halve again) or the two-minute treatment ended.

At the beginning of each experimental day, the transducers were positioned in degassed, deionized water and manually co-registered with the MRI scanner's isocenter by laser alignment with a fiducial marker. Anesthesia was induced with 2% isoflurane. During FUS treatments the carrier gas was changed to medical air, while for the remainder of the procedures, from preparation to sacrifice, isoflurane was dissolved in oxygen. The scalp was prepared following electric hair clipping and application of cream depilation (Veet; Reckitt Benckiser Group plc, UK) prior to acoustic coupling with ultrasound gel. Lastly, each rat was placed supine on a custom-made 3D-printed sled adapting to both the FUS and MRI animal holding systems. Intravenous (IV) injections were possible thanks to a catheter positioned in the tail vein (either 22G or 24G).

With the animal preparation complete, a pretreatment axial T1-weighted sequence was acquired. This ensured feasibility for brain treatment targeting and animal positioning. In tumor-bearing animals, to facilitate the visualization of the tumor volume, an additional pre-treatment T1-weighted (T1w) sequence was acquired following the administration of Gadolinium (0.1 ml/kg; Gadovist, Bayer Inc., Canada). This strategy leverages the inherent permeability of the blood-tumor barrier to distinguish the unaffected healthy GM and WM from the glioma.

The images were then loaded onto the LP-100 software to target the unilateral brain structures of interest. With such an approach, the contralateral untreated hemisphere served as an internal control for both MRI and fluorescence microscopy measurements.

In the healthy rat cohorts, the unilateral striatum, corpus callosum and internal capsule were targeted for FUS, resulting in a sonication grid ranging from 3 x 3 to 4 x 4 spots (1.5 mm spacing). The aforementioned structures together with the entire tumor volume were instead targeted in the glioma rat cohorts with a grid adapting to the anatomical targets, reaching sizes of up to 6 x 6 spots.

According to group allocation, the animals were then injected IV with either one of the antineoplastic drugs (bevacizumab or methotrexate), followed by Gadolinium and Definity microbubbles (MBs; 0.02 ml/kg, Lantheus Medical Imaging, USA).

MRI contrast deposition and treatment outcomes were monitored *in vivo* following each FUS delivery by means of serial post-treatment T1w sequences. A total of n =12 healthy animals, pre-assigned to a repeated FUS treatment strategy, underwent a second injection of gadolinium and microbubbles immediately prior to a second FUS sonication. Repeated FUS was administered 30 minutes after the first ultrasound exposure over the same gray and white matter structures.

### Tissue preparation and fluorescence microscopy

Two hours after the last received treatment, all animals were perfused with vascular dyes and sacrificed.

Bevacizumab-receiving rats were transcardially perfused with sequential administration of phosphate buffered saline (PBS), 10% formalin and lastly fluorescein isothiocyanate(FITC)-albumin conjugate gel. Based on prior protocols 70, the solution included 2% (w/v) porcine skin gelatin (Sigma-Aldrich, Catalog no. G2500) and 0.1% (w/v) FITC-albumin (Sigma-Aldrichm, Catalog no. A9771) dissolved in PBS. Gelatin and PBS were first mixed at room temperature, then brought to a boil. As the temperatures reached 60 °C, FITC-albumin was added, and the solution continuously mixed at 42 °C with a magnetic stirrer. Prior to transcardiac injection, the gel solution was filtered using 0.8 µm pore-size syringe filters (Corning, Catalog no. CLS431221). For the gel to solidify intravascularly, the animals were placed in ice for 30 min, then the brains were exercised for overnight fixation in 10% formalin.

In preparation for high-throughput light sheet fluorescence microscopy, samples require clearing, a process by which their refractive index (RI) matches the one of both the imaging medium and lenses; resulting in optically-transparency to the lasers positioned orthogonally to the camera sensor. To address the challenges posed for large samples like the rat brain, two equivalent techniques were implemented for tissue clearing.

Healthy rat brains originated from a previous study (n = 12) 23 and were included for higher magnification analysis. The brain quadrants (approximately 8x8x8 mm) covering the treated and contralateral untreated areas were sectioned and immersed in CUBIC (clear, unobstructed brain imaging cocktails and computational analysis) 71, a passive clearing solution requiring up to 30 days at room temperature for optimal sample penetration and full-thickness clearing. The CUBIC medium was renewed every week.

At the time of the tumor cohort, an active clearing system was available, enabling clearing of the whole brain: SHIELD/Clear+ (stabilization under harsh conditions via intramolecular epoxide linkages to prevent degradation, LifeCanvas Technologies) 72. Samples were first embedded into an epoxy solution (SHIELD preservation), next, multiple steps of stochastic electrophoretic detersion and delipidation were required for whole-brain clearing.

In preparation for scanning, specimens were mounted onto a holder with a clear silicone adhesive and immersed in RI-matching solutions: 65% v/v glycerol in ultrapure water (RI = 1.42) for the CUBIC-cleared samples, and a custom oil (RI = 1.52, Cargille Labs) for the SHIELD/Clear+ processed samples. Light sheet fluorescence microscopy was performed at a Miltenyi UltraMicroscope Blaze (Miltenyi Biotec) with a voxel resolution of 0.97x0.97x5µm with an RI-matching 4.0x/0.35 NA objective lens at 1.67 magnification. The vascular channel captured FITC-albumin gel fluorescence at 488/525±50nm (excitation/emission), while the drug channel captured the distribution of fluorescent conjugated bevacizumab at 640/680±30nm.

Prior experimental testing 23 found either of the two tissue clearing methods to remove methotrexate from the brain, potentially due to its small (454.44 Da) lipophilic structure. Therefore, methotrexate-receiving animals were perfused and processed for confocal microscopy following a distinct protocol.

An incision over the chest cavity was made, exposing the heart for *in vivo* catheterization under 5% isoflurane anesthesia. Firstly, the vascular endothelium was stained via a transcardiac injection of DyLight 649-conjugated Lycopersicon Esculentum Lectin (LEL) (Thermo Fisher, Catalog no. L32472) 73. The dye was administered over one minute and allowed to circulate in the bloodstream for 3 minutes prior to perfusion with ice cold saline. Collected brain samples were flash frozen in liquid nitrogen and stored at -80 °C, in preparation for cryosectioning at a cryostat (Leica CM3050S cryostat, Leica Biosystems) in consecutive 40 µm -thick slices. Specimens were transferred to charged microscope slides and fixed with neutral buffered formalin. Blocking was performed with 1% normal donkey serum (Jackson ImmunoResearch, Catalog No. 017-000-121) diluted in PBS. Slices were then incubated at room temperature for one hour with a primary anti-methotrexate sheep polyclonal antibody (Thermo Fischer Scientific, Catalog No. PA5-33142, dilution 1:100), washed five times via submersion in PBS and incubated again for one hour with a secondary donkey anti-sheep Alexa Fluor™ 488-conjugated antibody (Thermo Fischer Scientific, Catalog No. A-11015, dilution 1:500). Lastly, slides were wet mounted with a DABCO-based polyvinyl alcohol medium (Sigma-Aldrich, Catalog No. 10981) and coverslipped. The slides of the methotrexate-receiving healthy cohort (n = 12) originated from a previous study 23; included here with higher magnification imaging and analysis.

A confocal microscope (Nikon A1R) was used to acquire images of all treatment cohorts at a voxel resolution of 0.27x0.27x3.43µm with a 60x/0.36 NA objective lens at 1.72 magnification. Methotrexate fluorescence was detected at 488/525 ± 25 nm, while Dylight-conjugated LEL fluorescence was captured at 640/700±38nm, imaging the brain vasculature.

### Image Analysis

The T1w MRI scans underwent preprocessing of intensity non-uniformity normalization via N4 74, manual extraction of brain masks and gaussian filtering (radius: 2 voxels). Images were rigidly (3 Degree-Of-Freedom; DOF) co-registered to baseline acquisitions, then the MRI acquisition set of each animal was fed to a stepwise registration pipeline, from single animal space to a common Fischer-344 brain atlas space 75. Registration transforms were concatenated in a series of rigid (6 DOF), affine (12 DOF) and non-rigid warped symmetric diffeomorphic transformations 76.

While GM and WM tissue masks could be successfully obtained from the now-registered brain atlas segmentation, tumor volumes required semi-automatic contouring, guided by the Gadolinium contrast uptake on pretreatment images, prior to the registration pipeline. In post-treatment scans, Gadolinium enhancements aided in the manual definition of regions of interest (ROIs) exposed to FUS. Group differences were compared by means of percentage signal change between the treated and untreated (contralateral) sides of each tissue. Tumor tissues, implanted unilaterally, were instead compared to the contralateral untreated gray matter.

The channels acquired in fluorescence microscopy (bevacizumab and methotrexate-receiving cohorts) were first preprocessed separately, then merged for whole-volume serialized 2D colocalization analysis. Tissue masks were manually defined for gray matter, white matter and tumor (limited to the cellular component, excluding the necrotic core composed of proteinaceous fluid in a walled-off avascular acellular compartment) following anatomical boundaries evidenced by MRI and autofluorescence signal. The following microscopic measurements were independently assessed for each of the examined tissues.

Vascular channel images underwent rolling-ball background subtraction, contrast enhancement and gaussian filtering (radius: 2 voxels) prior to vascular whole-segment diameter estimation via Chamfer distance mapping 77, providing voxel-based measurements limited by image resolutions.

The frequency histograms of the vascular diameters present in each tissue were obtained, and blood vessels classified into capillaries (< 5 µm), microvessels (including arterioles and venules, 5-10 µm) and major vessels (>10 µm) 78-81. Vascular densities, expressed as ratio of intravascular volumes to total image sample, were also measured **(Figure 1B)**.

From the extravascular spaces, inter-vessel distances were quantified for each tissue and segmented into three components: perivascular (PV, radially extending by 50% of the vessel diameter) 82-84, 25% of total inter-vessel distance (IVD25) and 50% of total inter-vessel capillary distance (IVD50) **(Figure 1C)**.

Drug channel images underwent histogram normalization to the first slice of the stack, contrast enhancement and gaussian normalization (radius: 2 voxels). Intensity thresholds were used for distinguishing fluorescence signal peaks, related to drug extravasation, from the homogeneous background signal 85. An iterative thresholding algorithm including rolling-ball background estimation 86 and Otsu's thresholding 87 was separately applied to GM, WM and tumor tissues in order to account for the underlying signal intensities related to the distinct parenchymal architecture. Fluorescent drug intensities were measured for both intravascular and extravascular spaces of the vasculature tree as a whole, as well as divided by vessel diameters.

Vascular and drug channels were eventually merged to estimate the average distances between the extents of extravasated fluorescent drug signals and the nearest vessel lumen via geodesic distance mapping 88,89. The spatial distributions of drug intensities were then correlated with the average blood vessel diameters and the geodesic extravasation distances via Pearson's correlation coefficient.

### Statistical Analysis

The Benjamini-Hochberg method 90 was used to control the false discovery rate in this study's multiple hypothesis testing. The threshold for two-tailed statistical significance, rejecting the null hypothesis, was thus set to p < 0.034.

Within-animal comparisons among tissues were analysed using paired samples t-test. Between-animal comparisons for drug and FUS treatment strategy groups were instead analysed by univariate analysis of variance (ANOVA). Histograms were compared via Mann-Whitney U test and Kolmogorov-Smirnov test respectively for differences in central tendency and overall data distribution.

## Results

### MRI-guided focused ultrasound

The surgical implantation of F98-glioma cells in the deep gray matter was successful in all animals (n = 24). No procedural complications were noticed and no rat was sacrificed before the time point for FUS treatment set at 10 postoperative days. The mean tumor volume, as estimated on gadolinium-enhanced pre-FUS MRI, was 199.4±69.8 mm^3^ (range relative to total intracranial volume: 3.5 - 10.4%), of which the necrotic core occupied 6.4 - 22.8%. Gliomas exhibited phenotypical F98-cell growth 91 with clear transition between the necrotic acellular proteinaceous core and the highly-cellular periphery invading the adjacent GM and WM 92.

Tumor margins were not clearly visible on baseline T1w scans. Due to the compromised integrity of the blood-tumor barrier, gadolinium was injected IV to visualize the extent of the glioma on an additional set of pre-FUS contrast-enhanced T1w scans.

Brain tumors, together with the bordering gray and white matter, underwent either sham or single FUS exposure. The acoustic pressures at each sub-spot within the targeting grid was automatically adjusted by the controller, based on the ultraharmonic emissions of the oscillating microbubbles 69,93, leading to mean peak negative pressures of 158.0 ± 44 kPa during the steady state portion of the treatment, derated for transmission through rat skull assuming 73% transmission at 0.5 MHz 94.

**Figure 2:**
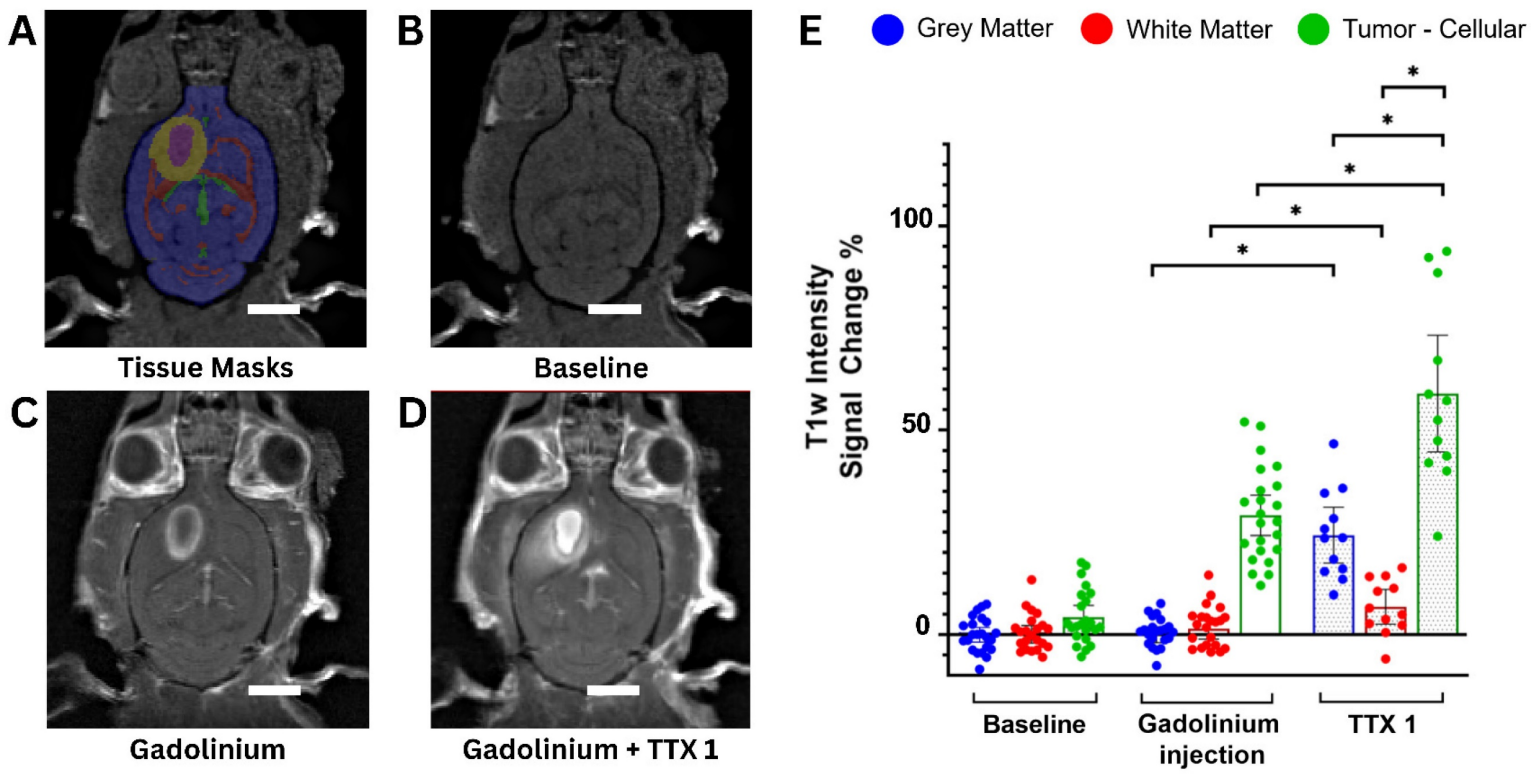
** (A-D)** Axial T1-weighted images of a Fischer-344 rat implanted with F98 rat glioma cells. The animal underwent serial scanning at baseline, before (A, B) and after (C) Gadolinium contrast agent injection, as well as following FUS treatment of tumor tissues (scale bar = 5 mm) (D). Tissue masks (A) were derived from a Fischer-344 brain atlas 75 and overlayed on images acquired at baseline, prior to any treatment showing gray matter (blue), white matter (red) and cerebrospinal fluid (green). The segmented tumor cellular compartment (yellow) and tumor necrotic core (purple) are also shown.** (E)** Gadolinium contrast enhancement in the tumor cohort expressed as percentage T1-weighted signal change compared to the contralateral untreated side (tumors were compared to the contralateral gray matter). A total of n = 24 tumor-bearing Fischer-344 rats underwent baseline imaging before and after intravenous MRI contrast injection, of which n = 12 animals underwent FUS and post-treatment imaging (TTX 1). For each animal, blue, red and green dots represent mean T1-weighted signal changes in the gray matter, white matter and tumor tissue, respectively.

Focused ultrasound treatments successfully increased the permeability of the brain vasculature in all rats, demonstrating increased gadolinium extravasation on post-sonication MRI in both the necrotic and cellular compartments **(Figure 2A-D)**.

On contrast-enhanced pre-treatment scans, the GM and WM did not show significant T1w signal changes compared to the contralateral side. However, in the cellular compartment of tumors it increased by 29.1 ± 11.4% (mean ± standard deviation). Following single sonication, signal changes were 24.2 ± 10.7% in the GM, 6.5 ± 6.64% in the WM and 58.9 ± 22.4% in tumor tissues (p < 0.001) **(Figure 2E)**.

The MRI results from the tumor-bearing animal cohort reveal statistically significant lower post-treatment signal changes in the WM compared to the GM, consistent previous findings in the healthy cohort 23.

### Drug deposition quantification

The microscopic images of both healthy and tumor-bearing cohorts were analysed for the assessment of drug delivery at the vascular level. Due to *ex vivo* tissue processing prior to imaging, the tumors' acellular necrotic core, constituted of proteinaceous fluid, dissolved during the washing and sectioning steps in the majority of the cases, irrespective of the administered drug molecule. Therefore, the analysis of fluorescence microscopy images focused solely on the cellular tumor compartments.

Bevacizumab and methotrexate signal intensities from the pre-processed drug channels were normalized to the adjacent tissue-specific background, as previously described, and expressed as fold change.

From sham to single FUS treatment, light sheet fluorescence microscopy of bevacizumab-receiving rats showed signal intensity was 1.04 ± 0.05 and 2.12 ± 0.64-times in the GM (p < 0.001), 1.03 ± 0.06 and 1.47 ± 0.36-times in the WM (p < 0.001), and 1.96 ± 0.43 and 3.70 ± 1.72-times in brain tumors (p< 0.001), compared to background.

**Figure 3:**
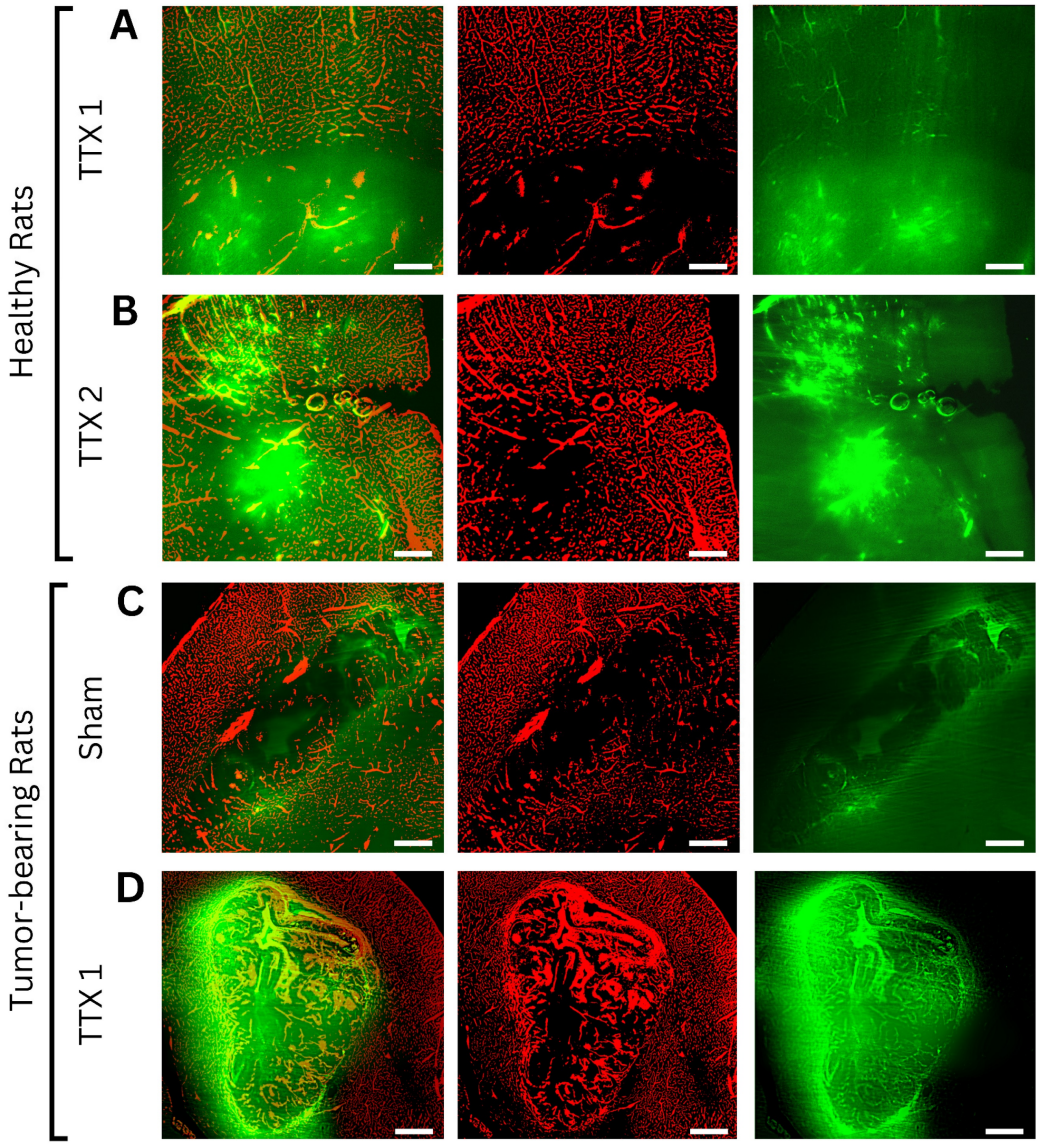
Axial light sheet fluorescence microscopy slices from whole-brain images of bevacizumab-receiving animals following **(A)** single and **(B)** repeated treatment in healthy rats, **(C)** sham and **(D)** single treatment in tumor-bearing rats (scale bar = 200µm). Each example consists of a composite image, followed by the vasculature channel (red) and the drug channel (green). Compared to gray matter, white matter and tumor tissues exhibit lower autofluorescence intensities, thus appearing darker regardless of drug extravasation. Bevacizumab deposition can be appreciated in areas of increased vascular permeability, both bound to vascular lumens and extravasated into the parenchyma. As bevacizumab cannot cross the intact blood-brain barrier (BBB), untreated healthy tissues show no signal enhancement. In contrast, tumors showed drug delivery regardless of FUS treatment, while ultrasound-exposed gray and white matter showed progressively higher intensities with each sonication.

**Figure 4:**
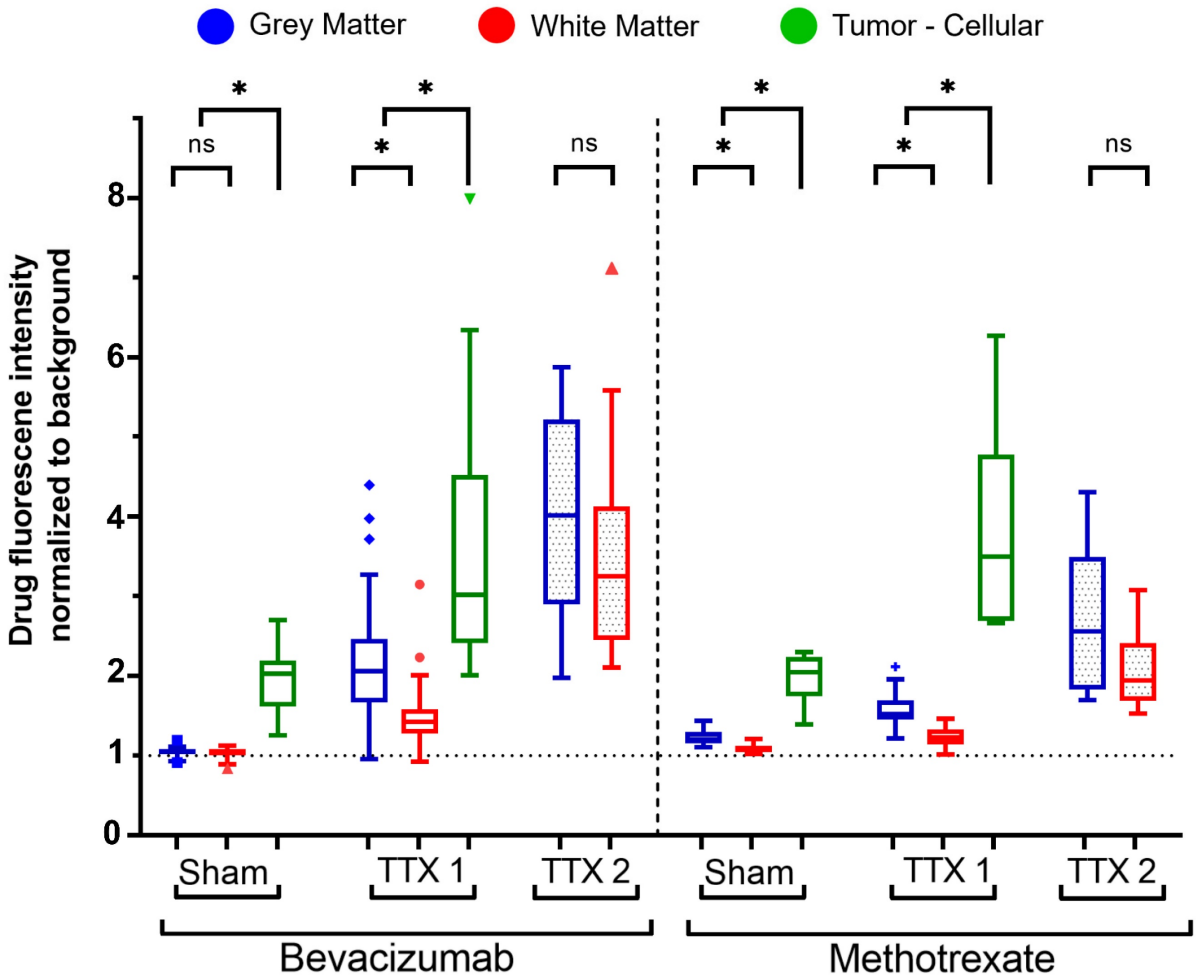
Fluorescence intensity quantification of bevacizumab (left) and methotrexate (right) normalized to background signals in healthy and tumor-bearing Fischer-344 rats undergoing sham sonication (n = 12 tumor-bearing rats), single FUS (TTX 1) (n = 12 healthy, n = 12 tumor-bearing rats), or repeated FUS (TTX 2) (n = 12 healthy rats) over the same unilateral targets. Analyses indicate tissue-type comparisons within each treatment group. Blue, red and green box plots represent mean fluorescence intensity fold changes respectively in the gray matter, white matter and tumor tissue.

Following a second sonication on healthy tissues, GM and WM enhancements respectively were 3.88 ± 1.22 and 3.48 ± 1.20-times the background (p < 0.001 for each tissue, when compared to fold changes in the single treatment group). Figures 3 and 4 indicate increased fluorescent signal uptake, hence bevacizumab deposition, following double permeabilization of the brain vasculature.

**Figure 5:**
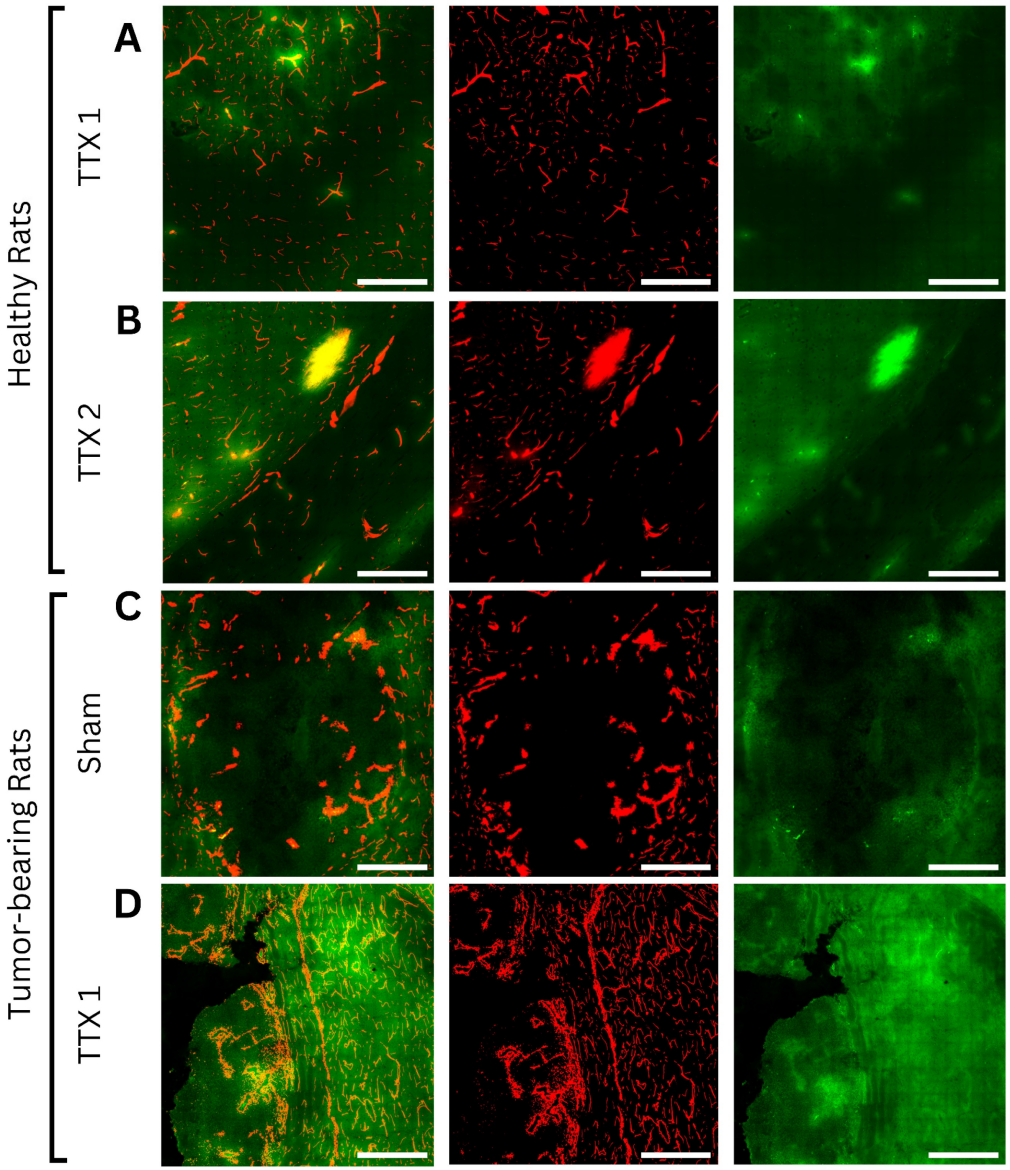
Axial confocal microscopy images of methotrexate-receiving animals following **(A)** single and **(B)** repeated treatment in healthy rats, **(C)** sham and **(D)** single treatment in tumor-bearing rats (scale bar = 200µm). Each example consists of a composite image, followed by the vasculature channel (red) and drug channel (green). Similar to bevacizumab-treated animals, white matter and tumor tissues exhibit lower autofluorescence intensities than gray matter, appearing darker regardless of drug extravasation. Due to its small molecular size and high lipophilicity, methotrexate can permeate both the physiological blood-brain barrier and pathological blood-tumor barrier, resulting in visible gradients of extravascular deposition in both treated and untreated regions. Drug signals progressively increased with each FUS treatment in both healthy and tumor tissues.

Similarly, confocal microscopy of methotrexate-receiving rats showed tissue-specific increases in fluorescence signals following FUS treatments **(Figure 4, 5)**. Signal intensity in the GM was 1.22 ± 0.09 and 1.58 ± 0.22-times respectively in the sham and single treatment conditions (p < 0.001) compared to background; in the WM 1.08 ± 0.052 and 1.23 ± 0.12-times (p< 0.001). Following a second sonication on healthy tissues, GM and WM enhancements respectively were 2.67 ± 0.92 and 2.05 ± 0.48-times relative to background (p < 0.001 for each tissue, when compared to fold changes in the single treatment group). In brain tumors, compared to background, methotrexate signal was 1.96 ± 0.31-times the background in the sham cohort and 3.77 ± 1.30-times in the single FUS cohort (p < 0.001).

Comparing the deposition of bevacizumab and methotrexate fluorescent-drug signal** (Figure 4)**, there were no statistically significant differences for tumor tissues following either sham (p = 0.967) or single treatment (p = 0.902).

In untreated healthy tissues, methotrexate signal, relative to background intensities, was higher than bevacizumab in the GM by 16.3 ± 2.8% (p < 0.001) and by 5.4 ± 3.0% (p < 0.001) in the WM.

The gray and white matter targeted for single FUS instead registered higher bevacizumab signal increase by 37.2 ± 15.5% (p < 0.001) and 21.55 ± 10.4% (p < 0.001) respectively in the GM and WM, while by 76.5 ± 39.1% (p < 0.001) and 66.2 ± 27.8% (p = 0.002) following repeated FUS as compared to the increase in methotrexate signal.

### Drug extravasation measurements

**Figure 6:**
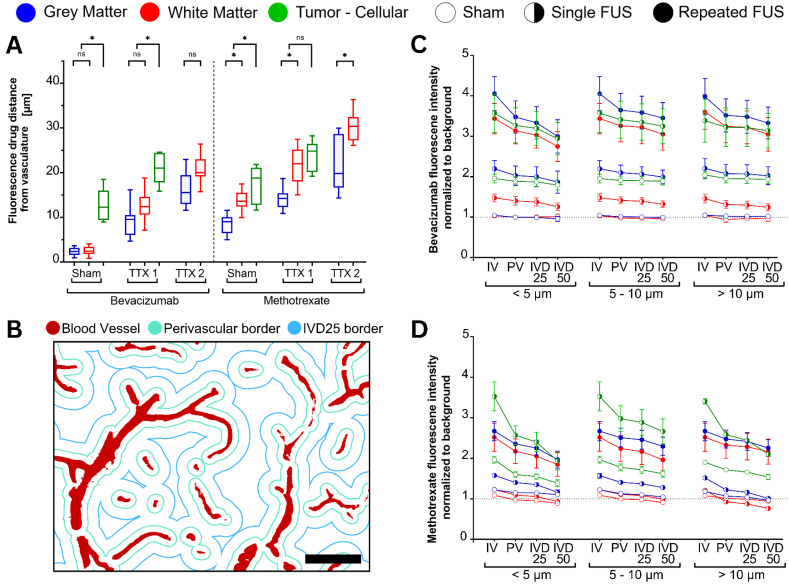
Fluorescence-based quantification of drug extravasation distance from the nearest blood vessel was performed for each tissue type and FUS treatment condition (total n = 48 animals, equally allocated by administered drug, FUS treatment strategy, healthy brain and tumor-bearing). **(A)** Average extravasation distances for bevacizumab (left) and methotrexate (right) following sham, single (TTX 1), and repeated (TTX 2) treatments. FUS progressively increased drug penetration into deeper interstitial spaces, with methotrexate showing the greatest extravasation distances. **(B-D)** Fluorescence intensity normalized to background signal in intravascular (IV) and extravascular compartments (B) (scale bar = 50µm) segmented into perivascular space (PV), 25% of inter-vessel distance (IVD25), and 50% of inter-vessel distance (IVD50), for both bevacizumab- (C) and methotrexate-receiving (D) animals. Blue, red, and green box plots represent measurements from gray matter, white matter, and tumor tissues, respectively. Darker shades indicate treatment conditions, while lighter shades represent sham controls.

With each FUS exposure, extravasation distances and delivery of both bevacizumab and methotrexate increased following tissue-specific patterns, while gradually decreasing at deeper inter-vessel compartments **(Figure 6)**.

The average distances recorded between blood vessels were 34.8 ± 10.6 µm in GM, 62.5 ± 22.9 µm in WM and 43.5 ± 21.0 µm in tumor tissues.

From the obtained signal and background masks, it was possible to estimate the average distances of extravascular drug distribution from the nearest vascular lumen **(Figure 6A)**.

Gray and white matter tissues exhibited bevacizumab extravasation distances respectively of 2.3 ± 0.9µm and 2.5 ± 0.9 µm (p = 0.334 between tissues) in untreated regions, significantly increasing to 9.0 ± 3.2 µm and 12.8 ± 3.0 µm (p < 0.001) following a first treatment. Upon repeated treatment, drug extravasations further increased to 16.2 ± 3.5 µm in the GM and 21.0 ± 3.1 µm in the WM (p < 0.001 between tissues). It should be noted that the higher vascular density of GM, and thus shorter distance between vessels, may account in part for this difference. Fluorescence signal is measured with respect to the closest vessel, and thus it is not possible to measure extravasation distance beyond the IVD50 (half the inter-vessel spacing) **(Figure 6B)**.

Similar to healthy tissue, tumor tissues showed increased bevacizumab extravasation following single-FUS (19.2 ± 3.0 µm), compared to sham (12.9 ± 5.2 µm; p = 0.013).

Within each treatment strategy prescribed per tissue, there were no statistically significant differences among the bevacizumab extravasation directly attributable to capillaries (diameter < 5 µm), microvessels (5-10 µm) or major vessels (> 10 µm) **(Figure 6C)**.

Intravascular drug signals, compared to the extravascular compartments were higher in all conditions, presumably due to both binding of BVZ to the endothelial cells and partial volume effects from the optical sectioning of the imaging instruments.

Proceeding from intravascular to half of the inter-vessel spaces (IVD50), signals decreased on average by 23.0 ± 10.0%, 11.7 ± 5.1% and 15.2 ± 5.0% respectively in capillaries, microvessels and major vessels of GM and WM receiving single treatment (p < 0.001 among vessels), compared to 43.3 ± 20.3%, 19.3 ± 7.9% and 23.5 ± 5.4% receiving repeated ultrasound (p = 0.697 among vessels, p = 0.707 between treatment groups).

In the same vessel categories, the drug signal in untreated tumors decreased by 10.7 ± 4.6%, 3.0 ± 2.0% and 7.0 ± 3.4% (p = 0.798), while in FUS single treated tumors they decreased by 23.1 ± 6.9%, 10.2 ± 7.5% and 6.1 ± 2.5% (p = 0.027, p = 0.001 between treatment groups) **(Figure 6C)**.

The histograms of bevacizumab extravasations from vascular spaces **(Figure S1)** showed right-skewed distributions for all tissues and treatments.

Following single and repeated treatments, the median extravasation distances of each tissue significantly increased from the untreated conditions with greater areas under the curve, reflecting the aforementioned results. The distribution of frequencies was also statistically different (p < 0.001), with peaks most evident at lower distances, possibly indicating greater numbers of vessels exhibiting extravasation with both single and repeated treatments. While in healthy tissues, frequencies rapidly decreased over greater inter-vessel space; in tumors, a plateau can be noticed, followed by a more gradual decrease. These observations, together with negative values of excess kurtosis, suggest greater spread of bevacizumab in tumor tissues, with evidence up to 60µm away from the nearest vessel.

The extravasated methotrexate signal **(Figure 6A)** was evident in the untreated conditions for GM (8.5 ± 2.2 µm), WM (13.9 ± 2.2 µm) and tumors (16.8 ± 4.7 µm). Following single-FUS, signal distances significantly increased compared to the sham condition for GM (14.3 ± 2.6µm; p < 0.001), WM (23.8 ± 5.6 µm; p < 0.001) and gliomas (24.9 ± 11.2 µm; p < 0.001). Following a repeated exposure, methotrexate extravasation distance significantly increased to 22.5 ± 6.2 µm (p < 0.001) in the GM, and 34.6 ± 5.4 µm (p < 0.001) in the WM.

Within each treatment strategy and tissue type, methotrexate extravasation showed no statistically significant differences among capillaries (diameter < 5 µm), microvessels (5-10 µm), or major vessels (> 10 µm) **(Figure 6D).**

With the increases in extravasation distances, the signal decreased by greater amounts. For single-treatment GM and WM, proceeding from intravascular to inter-vessel spaces, the signal decreased by 36.8 ± 11.0% in capillaries, 25.0 ± 5.1% in microvessels, and 55.1 ± 15.8% in major vessels (p = 0.006 among vessels). Following repeated sonication, these reductions were 40.3 ± 20.5%, 25.4 ± 12.2%, and 18.1 ± 5.4%, respectively (p = 0.001 among vessels, p < 0.001 between treatment groups).

In untreated tumors, methotrexate drug signal decreased with distance from vessels by 48.2 ± 14.2% in capillaries, 22.9 ± 12.1% in microvessels, and 27.0 ± 9.5% (p = 0.223). In contrast, FUS single-treated tumors exhibited more pronounced respective reductions of 106.5 ± 25.9%, 42.7 ± 18.7%, and 61.9 ± 12.2% (p = 0.030 among vessels, p < 0.001 between treatment groups) **(Figure 6D).**

The histograms of methotrexate extravasation distances **(Figure S2)** showed right-skewed distributions for all tissues and treatments. Following single and repeated treatments, the areas under the curve of GM, WM and gliomas significantly increased throughout the inter-vessel distances, indicating enhanced Methotrexate extravasation in the interstitial compartments.

Comparing the distributions of extravascular drug deposition between the two antineoplastics, MTX exhibited greater extravasation distances across all conditions compared to BVZ **(Figure 6A).** Bevacizumab distributions, instead, exhibited greater frequency peaks at shorter distances with each FUS sonication.

Combining GM and WM, the drug signal following single exposure decreased by 17.0 ± 22.1% between the intravascular compartment and IVD50 in BVZ-receiving animals, and by 36.2 ± 21.5% in MTX-receiving animals (p < 0.001). With a second treatment, these were 29.1 ± 38.1% and 27.9 ± 17.5% (p = 0.053). In tumor tissues instead, the signal decreased by 6.9 ± 9.6% in the BVZ sham condition and by 32.7 ± 31.4% in the MTX sham condition (p < 0.001). Following single treatment on tumors, they were 14.4 ± 13.5% and 70.4 ± 65.3% (p = 0.007)** (Figure 6C-D)**.

A general linear model (GLM) was used to investigate the relationship between extravasation intensities parametrized by tissue type (GM, WM, glioma), injected drug (BVZ, MTX) and number of treatments (sham, single, repeated). There was a statistically significant positive correlation with extravasation distances (p = 0.001; F = 11.43), with significant effects for the injected drug (p = 0.016, F = 5.84), and the interaction term for tissue types and number of treatments (p < 0.001.; F = 41.85). The interaction term between all effects was also significant (p = 0.003, F = 2.38). The overall model yielded an R^2^ of .810 (adjusted: .797) with greatest proportion of variance explained by tissue types and number of treatments, as indicated by the F-statistic.

### Vascular analysis

**Figure 7:**
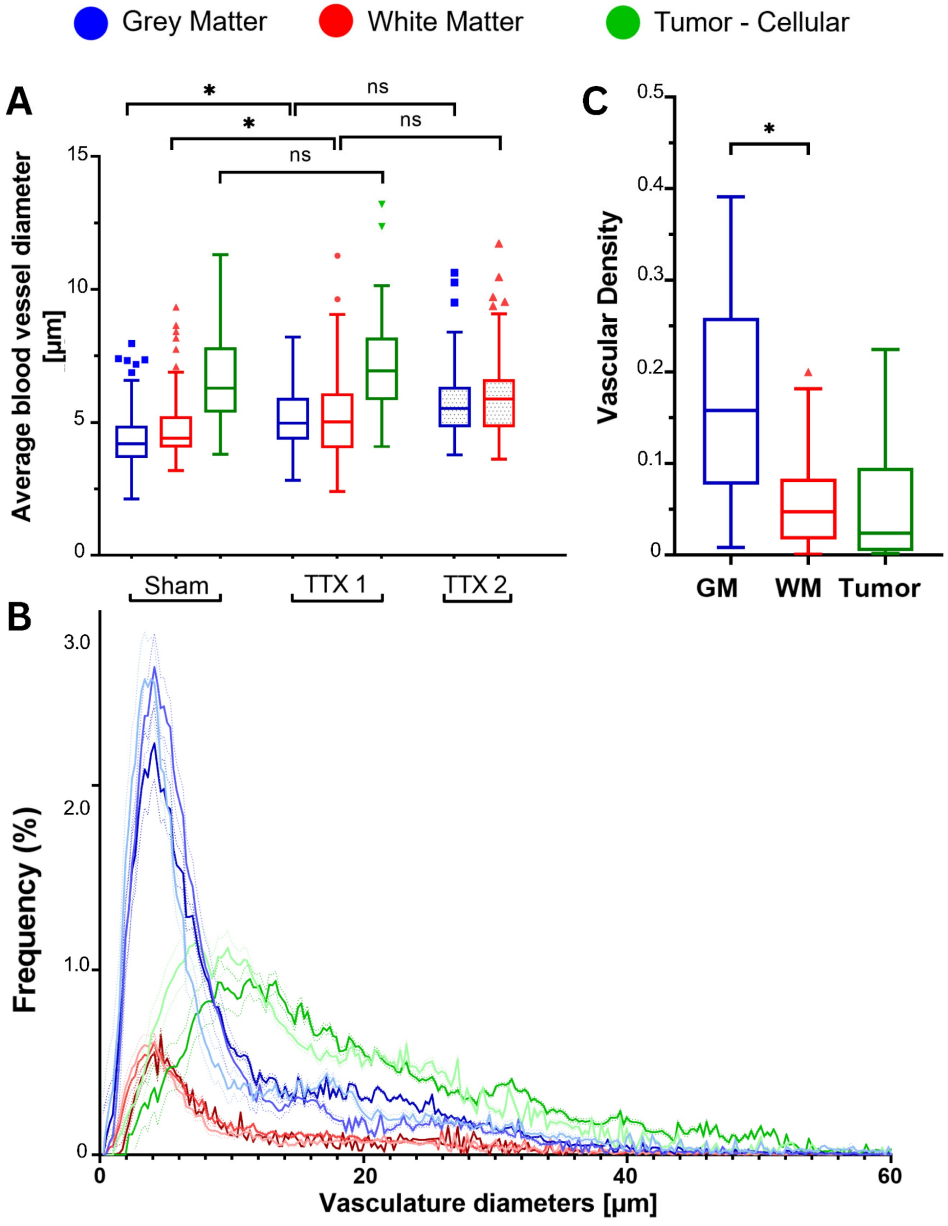
Fluorescently labeled blood vessels were analysed to determine vessel diameters and total intravascular area in each sample (total n = 48 animals, equally allocated by FUS treatment strategy, healthy brain and tumor-bearing). **(A)** Average vessel diameters in each tissue type following sham, single (TTX 1), and repeated (TTX 2) treatments. Gray and white matter exhibited increased mean vessel diameter following a single FUS exposure, with no further changes after repeated sonication, while the tumor vasculature remained unchanged. **(B)** Histograms of estimated vessel diameters, averaged across animals and organized by tissue type and treatment condition.**(C)** Tissue-specific vascular densities, calculated as the ratio of intravascular volume to total imaged sample volume. No differences were found between treatment conditions, while the fold change between tissues was consistently observed. Blue, red and green colors used in box plots and histograms indicate measurements obtained from gray matter, white matter and tumor tissues. In histograms, darker colors indicate treatment conditions, while lighter colors denote sham controls.

Segmented brain vasculature exhibited increased mean vessel diameter only in gray and white matter following single FUS, with no further changes after repeated sonication, while vascular density measures highlighted consistently higher values for GM (**Figure 7**).

Following a first treatment, mean blood vessel diameters increased from 4.4 ± 1.2 µm to 5.2 ± 1.4 µm in the GM (p=.002) and from 4.9 ± 1.3 µm to 5.4 ± 1.9 µm in the WM (p=.009). The additional increase in vessel diameters with a repeated exposure was not significant (GM: 5.9 ± 1.7; WM: 6.3 ± 2.0) **(Figure 7A)**.

In brain tumors, vascular diameters were 6.6 ± 1.8 in the sham condition and 7.4 ± 2.3 in the FUS condition (p = 0.629).

Overall, there were no differences between GM and WM for each treatment condition, while tumors exhibited greater vessel diameters (p < 0.001). No further differences were found when comparing the vasculature of animals injected with bevacizumab or methotrexate.

Specifically for brain capillaries, statistically significant differences were found only for the gray matter, with diameters increasing from 3.0 ± 0.5 in untreated areas to 3.1 ± 0.5 (p = 0.021) and 3.2 ± 0.5 (p = 0.011) respectively with single and repeated FUS **(Figure S3)**. Arterioles and venules exhibited differences only following a repeated treatment. Vessel diameters dilated from 5.86 ± 0.85 in the untreated GM to 6.0 ± 0.6 (p = 0.098) and 7.2 ± 1.4 (p <.001) with each sonication; and respectively from 5.9 ± 0.8 in the WM to 5.8 ± 1.1 (p = 0.937) and 7.1 ± 1.3 (p = 0.001).

Lastly, major blood vessels demonstrated significant differences only in the WM, with diameters of 15.6 ± 1.5 in the untreated condition, increasing only from 15.1 ± 2.0 following single-FUS to 16.7 ± 3.6 (p = 0.009) following repeated-FUS.

No differences were found for the tumor tissues in any of the vascular size groups **(Figure S3)**.

The histograms of vessel diameters showed right-skewed unimodal distributions for all tissue and treatment groups **(Figure 7B)**.

Following single and repeated treatments, there were no differences for the central tendencies of each examined tissue. Compared to untreated brain regions, however, their distributions indicate statistically significant shifts towards larger diameters with each ultrasound exposure. Specifically, the median for the untreated GM was 5.8 (IQR: 3.4 - 16.0), while 6.6 (IQR = 3.9 - 12.9) after single-FUS and 7.0 (IQR = 3.9 - 15.7) after repeated-FUS. In the WM they were respectively 6.1 (IQR = 3.6 - 17.0), 7.3 (IQR = 4.1 - 16.7) and 7.5 (IQR = 4.6 - 18.2). The medians for sham and treated tumors were 13.6 (IQR = 8.0 - 23.0) and 16.7 (IQR =10.7 - 27.1).

The analysis of vascular densities highlighted the intrinsically distinct architectures of GM, WM and gliomas **(Figure 7C)**, showing, as expected, non significant changes with treatments **(Figure S4)**. Vascular densities in the GM were higher than in the WM (3.2 ± 0.8-folds; p < 0.001) and in tumors (3.1 ± 1.0-folds; p < 0.001). White matter and gliomatous tissues instead showed no significant differences in densities (p = 0.739). These results reflect the larger inter-vessel distances found in WM and tumors, compared to GM.

Lastly, a GLM was used to assess the effect of tissue-specific vascular densities, number of FUS treatments and injected drug molecule on drug extravasation distances with an R^2^ = .647 (adjusted: 0.605) with greatest proportion of variance explained by the administered drug, as indicated by the following F-statistics.

Significant main effects were observed for drug type (p < 0.001; F = 64.41) and interaction term for tissue type and number of treatments (p < 0.001; F = 5.74). Positive correlations were found with extravasated signal intensity (p = 0.038; F = 4.32), and negative with vascular densities (p < 0.001; F = 4.57), explained by the greater inter-vessel distances of WM and tumors.

## Discussion

In this study we assessed drug extravasation in terms of fluorescence intensities and distances at the vascular level following single and repeated feedback-controlled FUS-mediated modulation of the blood-brain barrier in three distinct tissue architectures: gray matter, white matter and tumor.

The gray and white matter are the two major anatomical components of the central nervous system in physiological conditions, featuring active filtration of molecules through the BBB. Gliomas are characterized by underlying inflammatory processes and vascular re-organization, leading to a leakier, less efficient barrier, the BTB.

Here, we investigated drug delivery across three states of barrier permeabilization: impermeable, single permeable, typical of healthy tissues undergoing single FUS treatment and untreated tumors; and double permeable, observed in healthy tissues undergoing repeated FUS and in tumors receiving single treatment. Importantly, the results of this study provide evidence that at a single-vessel level vessel permeabilization resulting from FUS and microbubbles varies grey matter, white matter and tumors. This is an important finding as grey matter and white matter differences in healthy tissues have long been attributed to differences in vascular density between these tissues.

Focused ultrasound exposures repeated at 30-minutes intervals are a promising treatment strategy to further increase the permeability of said blood barriers and deliver higher drug concentrations compared to single exposures 23. A vessel-level analysis of FUS-induced drug extravasation was performed in this study following previously described MRI-guided treatment and microscopy methodologies 23 with tissue comparisons extended to F98 gliomas.

Starting with ultrasound treatments, we used a feedback-controlled algorithm 69 based on ultraharmonic bubble emissions. The average acoustic pressure, derated for skull transmission 94, throughout the 2-minute treatment duration was ≈158.0 kPa when targeting tumors, while in the healthy animal cohort, targeting gray and white matter 23, pressures were ≈145.1 kPa following a single sonication, decreasing to ≈131.8 kPa following a repeated sonication. Such a difference may suggest that both the status of increased vascular permeabilization and the larger mean vessel size found in gliomas affect microbubble behaviour, starting to exhibit unstable cavitation at lower pressures. Considering the full width half maximum of the ultrasound beam generated by the described preclinical rat system, the axial focus region spanned across the entirety of tumor tissues. In the present study, the focus overlapping both GM and WM compartments was an advantage as it ensured consistent exposures between the two tissues, allowing for direct comparison. As for the safety of such treatment strategies, petechiae were found to correlate with higher ultrasound pressure as early as after single treatment, rather than with repeated sonication, as evidenced in rats and pigs 23,56,57 via *in vivo* T2* MRI sequences and *ex vivo* microscopy. Still, an in-depth safety assessment is needed in future studies, particularly to evaluate the long-term effects on sterile inflammation and the neurovascular unit 13.

While the distinct cellular architectures among GM, WM and tumors can affect acoustic transmission 95,96, arguably more important aspects to consider for microbubble-mediated ultrasound treatments are the underlying permeability status and arrangement of the vascular tree. The white matter is composed of a vascular organization equivalent to the one in the gray matter but with fewer blood vessels 28, while brain cancers, particularly high-grade gliomas 97, feature heterogeneous vascularity both within tumor regions and between tumor phenotypes, posing additional diagnostic and prognostic challenges 98,99. Here, we reported that GM is ≈3.17 times more vascularized than WM, in concordance with previously described values ranging 2 to 4 times across animal species 27,100. Similarly, F98 gliomas showed ≈3.11 times lower vascular density than the GM, reflecting their genetic predilection for vascular co-option despite inoculation site. As evidenced by histograms, the distribution of vessel diameters was similar between healthy tissues, however tumors showed values skewed towards greater sizes, with approximately double the median diameters. Such differences in vascular populations may be attributable to co-option, a phenomenon where, in order to meet metabolic demands, rapid tumor growth leads primarily to vessel enlargement 101,102. At a macroscopic level, as more blood vessels are present in a given tissue, more bubbles have the opportunity to interact with ultrasound exposures, resulting in overall greater and more homogeneous drug deposition 23. At the level of single blood vessels, however, drug extravasation distances and intensities were found dependent on the number of ultrasound treatments, injected drug and tissue types with weaker negative correlations with vascular densities. As different brain architectures feature unique cellularities, vascular densities** (Figures 7, S4)** and diameter distributions **(Figures 7, S3)**, it is critical to assess their impact for the design and optimization of tissue-specific treatment strategies.

In particular for tumors exhibiting co-option such as F98 tumors 89,90, greater vascular diameters may pose fewer constraints on microbubble sizes, with ultrasound inducing cavitation in bubbles with greater radii to start with 103-105. Focused ultrasound-mediated modulation of the vascular permeability has been shown to be effective and reproducible with a number of microbubble formulations, both monodisperse, characterized by homogeneous size-limited diameters 106-108, and polydisperse 109,110, presenting a wide range of sizes. In this body of work we used Definity microbubbles, a polydisperse formulation with diameters ranging from below 1 μm to above 10 μm 109. In light of the presented results, future research should focus on assessing the impact of bubble sizes on cavitation occurring at the vascular-levels of different brain tissues 106.

In FUS treated animals, mean vessel diameter was significantly larger than the untreated condition in both GM and WM, with non-significant additional increases following a repeated exposure. The leaky tumor vasculature did not experience diameter changes at the 2-hour sacrifice timepoint. Capillaries and arterioles are known from in-vivo fluorescence studies to undergo vasospastic responses for up to 15 minutes post treatment 12,14 with periodic vasoconstriction and vasodilation possibly as an attempt to mitigate the increased transport of interstitial fluid across the BBB. The results presented in this manuscript represent an ex-vivo assessment of the brain vasculature two hours post treatment, following animal perfusion and decapitation, with no differences in vessel diameter between the two tissue processing techniques used for methotrexate- and bevacizumab-receiving cohorts. The increased vascular diameters following FUS may therefore reflect a condition of augmented vascular compliance, more than a long-lasting vasodilation. Such a change in vascular compliance could also partially explain the difference in treatment pressures reached by the controller between the first and second treatments, however additional investigation would be necessary to confirm such a hypothesis.

On contrast-enhanced MRI images, GM and WM did not show significant T1w signal changes compared to the contralateral side, as expected, whereas tumors exhibited a marked increase due to the underlying compromised integrity of the BTB. Following a single sonication, the signal change was more pronounced in the GM than in the WM, with tumors displaying the highest increase. Compared to a previously studied healthy rat cohort 23, baseline tumor enhancement was similar to the contrast uptake observed in the GM after a single treatment, while the tumor response to sonication corresponded to the permeability levels measured in both GM and WM following repeated exposure. Contrast enhancements thus appear to correlate with tissue types and their underlying permeabilization, irrespective of the number of gadolinium injections 23. Such differences in the status of the brain barriers motivated further assessment of drug deposition with fluorescence microscopy.

In bevacizumab-receiving animals, light-sheet microscopy revealed signal increases in all tissues following sonication, with the highest enhancement in tumors. A second sonication in healthy tissues further amplified GM and WM deposition, reaching levels comparable to single-treated tumors. Methotrexate-receiving animals also showed signal increases across all regions, with tumors exhibiting the most pronounced enhancement.

Overall, drug deposition across treatment groups and tissue types showed similar trends to contrast-enhanced MRI, with the difference that methotrexate was found to permeate also the untreated BBB of healthy tissues.

Drug signal was highest in the intravascular spaces, progressively decreasing with distance from vascular lumens, demonstrating extravasation of the IV injected antineoplastics. All three diameter-defined blood vessel categories (capillaries, microvessels, major vessels) showed evidence of both drugs entering the interstitial spaces, albeit the drop in signal was more pronounced in larger vessels compared to capillaries, and for methotrexate compared to bevacizumab. A more accurate classification for the brain microvasculature should follow the branching number from major vessels, however would require specific arterial and venous staining 112,113. Other studies have shown dye extravasation increases as a function of blood vessel diameter, as evidenced in both capillaries and microvessels up to 10 μm with effective sonications following the administration of either microbubble or nanoscale bubble formulations 106,107,114.

In light of the most recent findings, FUS-mediated drug delivery appears to influence the permeability of not only the BBB and BTB, which are primarily associated with the capillary network, but it also extends to larger-caliber vessels 23, an observation consistent with early studies 115,116. While vascular densities related to neuronal presence are relatively maintained across mammals, despite differences among brain regions, vessel diameters and number of protective layers scale with brain sizes 117. While, in rats, major muscular arteries like the middle cerebral artery measure approximately 0.3 mm 118, arterioles and capillaries respectively range 5-10 µm 81 and lower than 5 µm 78-80. In humans, vascular diameters are greater, with middle cerebral arteries ranging 2-5 mm 119, arterioles averaging 10-20 µm 120 up to 50 µm 121 and capillaries lower than 10 µm 122. Thus, in larger animals and humans, considering vessel sizes and wall compositions, FUS-induced vascular permeabilization may be limited to local capillaries, arterioles and venules, while not extending to major regional and segmental arteries.

Drug extravasation was estimated based on tissue-derived intensity thresholds reflecting FUS-induced drug deposition between adjacent vessels. In bevacizumab-receiving animals, tumor tissues consistently showed greater extravasation distances compared to the GM and WM, which did not differ significantly from each other. In contrast, within the methotrexate cohort, both WM and tumors showed greater extravasation distances compared to the GM. This pattern may be attributed to the greater inter-vessel spaces available for drug diffusion due to the lower vascular densities of these tissues. When comparing the extravasation trends observed for both antineoplastics, it can be hypothesized that the differences in molecular weight played a more significant role than tissue-specific cellular density and architecture in determining their extravasation patterns. However, factors such as half-life, abundance of molecular targets and mechanisms of action, may still affect these results.

Analysis of the extravasation histograms revealed a progressive skewness toward greater distances with each successive sonication for both bevacizumab and methotrexate. Regardless of tissue type, in brain regions with a pre-existing permeabilized barrier, bevacizumab extravasation was most pronounced over short distances, suggesting an increased number of permeabilized vessels. Conversely, methotrexate exhibited greater benefit over longer distances, extending its distribution across the entirety of the inter-vessel spaces.

For both therapeutic molecules, at the single vessel level the drug signal intensity was lower for WM than GM, indicating that differences observed in drug delivery to the two tissues is not completely explained by vessel density. This suggests that the composition of both the BBB, BTB and endothelium may affect drug extravasation across tissues 29-32 and that repeated FUS exposures may overcome such disparities leading to overall greater pharmacological delivery 23.

The presented results are affected by methodological limitations that should be considered in future investigations.

While vascular lumens were segmented with the aid of specific dyes, extravascular spaces were defined according to iterative thresholding strategies 86 based on the estimated homogeneity of background signals. Bevacizumab cannot permeate the intact BBB of GM and WM, while only to a lower extent the leakier BTB. Methotrexate, instead, is able to cross the barrier of untreated tissues, both physiological and tumor ones. Therefore, the employed thresholding strategy could only separate the signal intensities attributable to FUS bioeffects: BVZ extravasation from tissue autofluorescence, and MTX delivery from a combination of tissue autofluorescence and low interstitial levels of systemically circulating drug. Additionally, given the slice thickness in the images, some signal classified as intravascular may in fact be from the perivascular space.

Focused ultrasound-induced drug delivery has been successfully employed in several rat models of both brain 66,68,120 and spinal cord tumors 52, however distinct cellular architectures, growth patterns and vascular compositions 91 are believed to affect treatment outcomes. F98 gliomas are composed of both necrotic acellular regions and hypoxic regions populated by rapidly dividing cells supplied by pre-existent vessels undergoing co-option. RG2 tumors as well as breast cancer-derived leptomeningeal metastases 123 constitute additional valuable tumor models, as they exhibit slower growths, lower cellular densities and highly angiogenic profiles, providing greater potential for anti-angiogenic therapies like BVZ and surface area for microbubble-mediated vascular bioeffects.

On post-treatment MRI, the necrotic core of F98 gliomas appeared hyperintense compared to both the surrounding cellular tumor component and the surrounding healthy brain. This can be explained by the accumulation of contrast agent within the acellular proteinaceous fluid 91,92 following vascular extravasation. A similar phenomenon was noticed in the BVZ cohort, with greater drug depositions in the necrotic core; however, this was not observed in the MTX cohort possibly due to the processing steps of sectioning and washing required for confocal microscopy

The tumor-involved samples planned for light sheet fluorescence microscopy underwent SHIELD/Clear+ tissue protection and clearing, an electrophoretically active methodology distinct from CUBIC used on previously studied samples 23,71. Although no differences in image clarity were observed, SHIELD/Clear+ enabled whole-brain clearing and imaging, without the need for quadrant sectioning.

Treatment protocols involving repeated FUS-mediated drug delivery demonstrate potential for clinical adaptation.

High-grade and diffusely infiltrating low-grade gliomas respectively represent 49% and 30% of all malignant brain tumors 124 associated with a 5-year survival ranging 5-45% with currently available treatments 125. Small molecule therapeutics like temozolomide and lomustine, as well as antivascular monoclonal antibodies like bevacizumab, coupled with surgical excision and radiation therapy constitute the current standards of care 125, 126. As new actionable cancer markers are being discovered; antibodies, immunotherapies and nanoparticles represent an area of increasing interest, with focused ultrasound potentially increasing local drug deposition. Gray and white matter therapies could similarly benefit from these techniques as novel poorly permeable monoclonal antibodies are being designed for neurodegenerative disorders 127, traumatic brain injuries 128, multiple sclerosis 129, and psychiatric illnesses 61,130.

Considering that therapeutic efficacy may depend on the timing and concentrations of systemically injected therapeutics, repeated FUS treatments could be a viable strategy to modulate the vascular permeability across different tissue types allowing for even greater *in situ* depositions. This could reduce the need for multiple administrations in the short term and prevent dose-related systemic side effects. We note a limitation of the current study is that the dose dependency of the achieved drug distribution was not investigated, and should be examined in future work.

Still, there is a need to define the microbubble-mediated bioeffects, particularly considering the challenges of tissue-specific responses and the underlying vascular permeability status in consideration of the interval between prescribed treatment. Repeating ultrasound exposures after 30 minutes may amplify the effects of a first FUS exposure by acting on “primed” barrier-impermeable blood vessels undergoing post-treatment vasospastic oscillations. Microbubble oscillations therefore likely induce further displacement of the blood barrier components 131,132 and enhance the dispersion of drug molecules. Identifying the optimal interval for repeated treatment may require additional studies characterizing permeabilization, but also comprehensively characterizing safety.

In healthy barrier-impermeable tissues, drug delivery at the single-vessel level became comparable between GM and WM after a second treatment. However, following a single treatment, GM exhibited a higher signal concentration than WM. Possible explanations in this behavior include fundamental differences in vascular structures or blood flow conditions influencing microbubble behaviour. While capillary and arteriole lumen sizes are similar in both tissue types, WM presents higher expression of tight junction proteins like claudin-5 and occludin 30 as well as thicker basal laminae 133, contributing to reduced permeability. Additionally, WM exhibits higher capillary blood flow in resting conditions, but delayed and less efficient neurovascular coupling, making it more susceptible to metabolic depletion 134. Evidence suggests that vascular flow influences sonoporation efficiency depending on drug molecular weight, though this has not been studied at capillary flow rates 135. Similarly, the choice of pulse repetition frequency relative to the reperfusion time will also influence the efficiency of FUS treatments 136. Lastly, the distinct composition of extracellular matrix proteins and lipids contributes to tissue-specific water retention and viscoelasticity 137, potentially influencing interstitial pressure, while perineuronal nets, cellular elements, and vascular basement membranes define their characteristic architectures 137,138.

In conclusion, our findings demonstrate that repeated FUS-mediated modulation of the intact BBB enhances drug deposition in both gray and white matter despite different tissue organization and vascularization. Drug signal intensities and extravasation measures were comparable to the levels obtained in rat brain gliomas following single FUS treatments, highlighting the potential of this strategy to further enhance drug delivery on already permeabilized brain tissues. Both small and large molecular size therapeutics exhibited similar trends with treatment bioeffects across capillaries, microvessels and major brain vessels. Vessel-level analyses show that differences in drug delivery to GM/WM is not fully determined by vascular density. Focused ultrasound-mediated drug delivery has been shown to interact with both administered antineoplastics and tissue types, with molecular gradients increasing in concentration and distribution over greater distances as more treatments further enhance blood-brain barrier permeability, despite differences in vascular density. Repeating FUS exposures to the BTB of brain tumor tissues thus represents an area of potential exploration to further enhance drug deposition and improve clinical outcomes. Diverse inter-sonication intervals have been investigated in both preclinical 41-44 and clinical 24, 43, 45 brain tumor studies, with treatment sessions scheduled from days to weeks apart, reflecting current standards of chemotherapy cycles. One study 139, in particular, found that FUS exposures repeated in the short-term, following a 20-minute interval, significantly increased deposition of both targeted and untargeted liposomal doxorubicin, decreasing glioblastoma tumor volume while enabling the administration of lower total drug doses to reduce systemic toxicity. Notably, while the pharmacochemical modifications of targeted doxorubicin improved biodistribution within tumor microenvironments in the absence of FUS 7,42,55, repeated ultrasound-mediated vascular permeabilizeation yielded similar *in situ* concentrations for both antineoplastics. Given the differential vascular architectures of healthy and pathological tissues, optimizing tissue-specific feedback-controlled ultrasound parameters could maximize treatment efficacy. Additionally, incorporating tissue-specific architectures and vascular features into biophysical models may improve the accuracy of outcome prediction 46,2. Future studies should investigate the long-term impact of repeated exposures, their efficacy on diverse tumor phenotypes and their long-term impacts on both toxicity and survival.

## Supplementary Material

Supplementary figures.

## Figures and Tables

**Table 1:** Animal cohorts distribution. BVZ = bevacizumab, MTX = Methotrexate, FITC = fluorescein isothiocyanate, LEL = Lycopersicon Esculentum Lectin

Fischer-344 Rat (200-250 g, male and female)	Number of Animals	Administered Drug	Treatment scheme	Staining	Fluorescence Microscopy
**Healthy**	6	BVZ (50 mg/kg)	Single	Vasculature: FITC-albumin gel Drug labelling: BVZ-conjugated Alexa Fluor™ 647	Light sheet fluorescence microscopy (LSFM)
	6		Repeated		
**Surgically implanted with F98-glioma**	6		Sham		
	6		Single		
**Healthy**	6	MTX (30 mg/kg)	Single	Vasculature: DyLight 649-conjugated LEL Drug labelling: anti-MTX antibody + Alexa Fluor™ 488	Confocal microscopy (CM)
	6		Repeated		
**Surgically implanted with F98-glioma**	6		Sham		
	6		Single		

## Abbreviations

FUS: focused ultrasound
WM: white matter
GM: gray matter
CNS: central nervous system
MRI: magnetic resonance imaging
BBB: blood-brain barrier
BTB: blood-tumor barrier
MTX: methotrexate
BVZ: bevacizumab
CM: confocal microscopy
LSFM: light sheet fluorescence microscopy
VEGF: vascular endothelial growth factor
IV: intravenous
MBs: microbubbles
FITC: Fluorescein isothiocyanate
ROIs: regions of interest
TTX: treatment
