# Supplementary material for: Tissue-type Differences in Focused Ultrasound and Microbubble-mediated Drug Delivery to the Brain Exist at Vessel Level
Source: Theranostics. 2026 Jan 1;16(4):1975–96. doi: 10.7150/thno.117691 (PMC12680594; doi:10.7150/thno.117691)
Supplement: Supplementary file 1 — Supplementary figures. [file thnov16p1975s1.pdf]

Supplementary

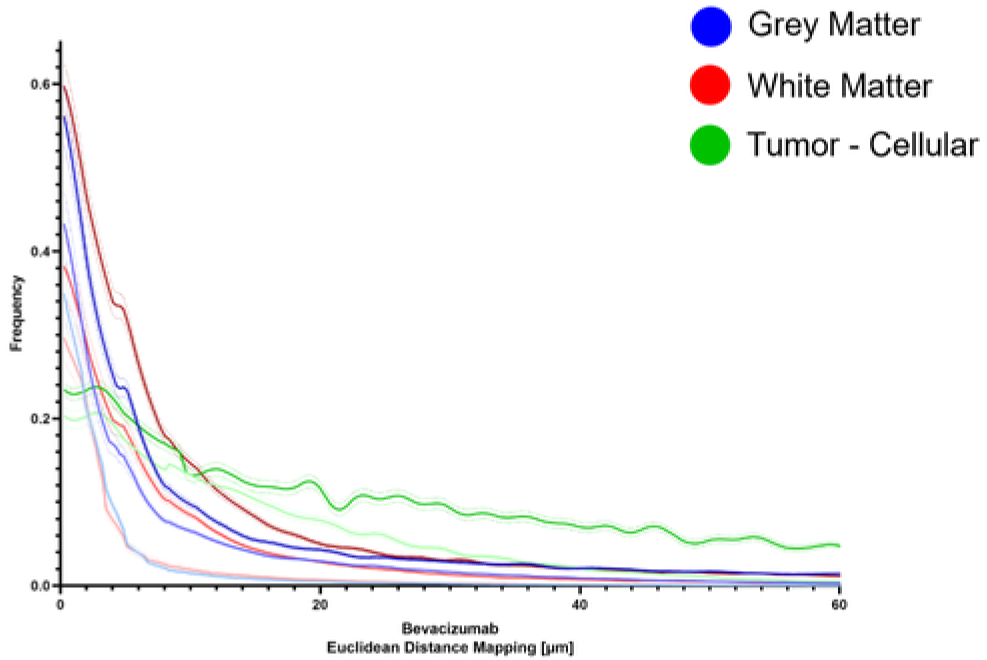

- **Supplementary Figure 1**

Bevacizumab extravasation distances, measured from the nearest blood vessel, were categorized and plotted as frequency histograms.
Blue, red, and green lines represent the percentage distribution of extravasation distances in gray matter, white matter, and tumor tissues, respectively. Darker colors indicate treatment conditions, while lighter colors represent sham controls.

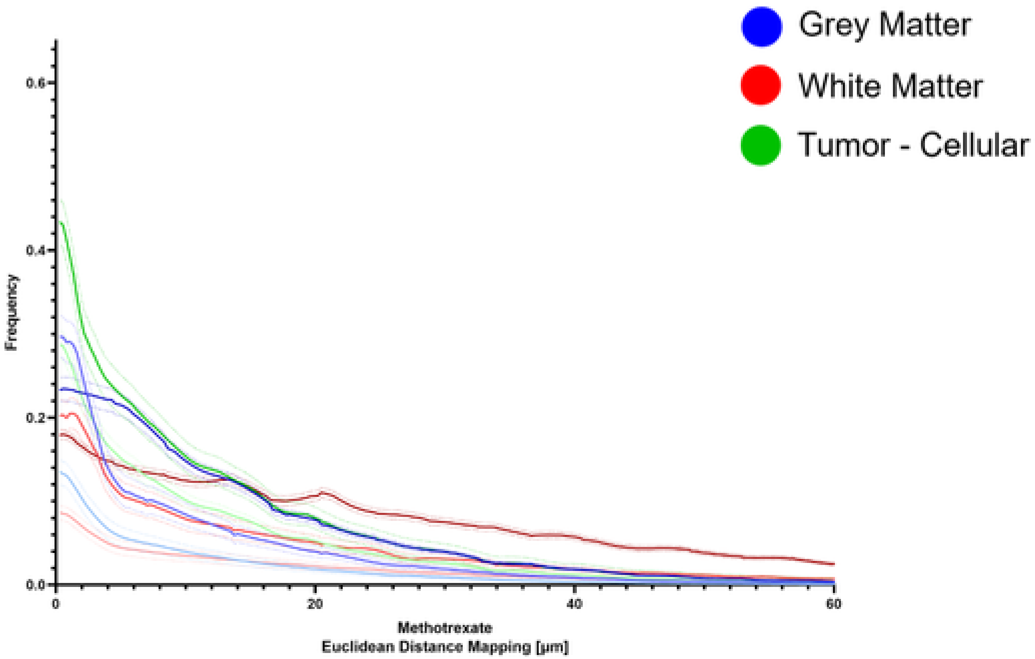

Supplementary Figure 2
Methotrexate extravasation distances, measured from the nearest blood vessel, were categorized and plotted as frequency histograms.
Blue, red, and green lines represent the percentage distribution of extravasation distances in gray matter, white matter, and tumor tissues, respectively. Darker colors indicate treatment conditions, while lighter colors represent sham controls.

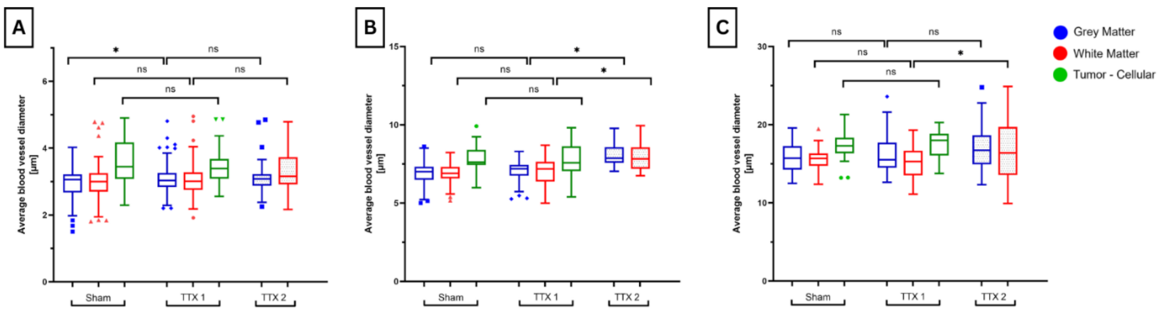

Supplementary Figure 3
Blood vessel diameters were categorized into capillaries (<5 µm), microvessels (5–10 µm), and major vessels (>10 µm). Single FUS treatments predominantly increased capillary diameters in the gray matter, while repeated treatments primarily affected microvessels and larger vessels in the white matter. Tumor vasculature remained unchanged.
Blue, red, and green box plots represent vessel diameters in gray matter, white matter, and tumor tissues, respectively.

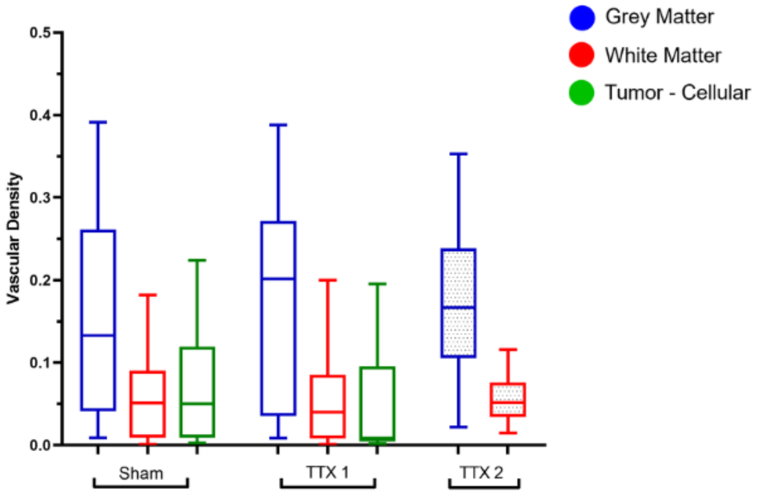

- **Supplementary Figure 4**

Vascular density calculated from the volume of intravascular spaces, directly estimated from the fluorescently labeled vasculature, was used to determine tissue-specific vascular densities. No significant differences were observed across treatment conditions, while the fold change between tissues was consistently observed.

Blue, red, and green box plots represent vascular densities in gray matter, white matter, and tumor tissues, respectively.
